
JOURNAL INFORMATION
==============================
NLM Title Abbreviation: Tex Med J (Austin)
NLM Journal ID: 21130590R

Texas Medical Journal

ISSN: 0892-8495
Publisher: Biomedical Journal Digitization Project

ARTICLE INFORMATION
==============================
PMCID: PMC9595681
Article version: 1
Subjects: Books and Magazines

Treatise on the Diseases of Women

For the Use of Students and Practitioners

Print publication date: 1899 Jul
Volume: 15
Issue: 1
First page: 54
Last page: 54
License: This work is free of known copyright restrictions. For more information, please see PMC Back Issue Digitization.
License URL: https://creativecommons.org/publicdomain/mark/1.0/

==============================

Treatise on the Diseases of Women. For the Use of Students and Practitioners. By Alexander J. C. Skene, M. D., LL. D., Professor of Gynecology in the Long Island College Hospital, Brooklyn, New York; President of the American Gynecological Association, 1887; Corresponding Member of the British, Boston and Detroit Gynecological Societies, etc., etc. Third edition, revised and enlarged. Fully illustrated. Published by D. Appleton & Co., N. Y. 1898. Pages, 990. Price, cloth, $6; sheep, $7. By subscription only.

No gynecologist stands higher in the ranks of progress today than Prof. Skene. His work has been recognized as one of the standards since its first edition. The author has kept abreast of the progress in his specialty, and brings to his work in preparing this third edition erudition and experience granted to but few men. That his readers gain the full benefit of these in the present volume, only a cursory reading is necessary to demonstrate. The volume is large, and nothing has been omitted that is necessary to make it a complete text-book.

Some of the improvements over former editions are, a clear and comprehensive description of the use of the endoscope and cystoscope, the control of hemorrhage by compression and electric heat, virtually doing away with the ligature, vaginal and abdominal hysterectomy have been brought thoroughly up to date, and many original and valuable procedures invented by the author thrown into almost every subject. The work is doubtless a safe guide, and is recommended heartily. J.

---

### Publisher's Department.

---

Sharp & Dohme have added two new pharmaceutical preparations to their well known list of specialties (Hypodermic Tablets, Pil. Lapactic, Ergotole, etc.), towit: Lithiated Sorghum Comp. (diruetic, demulcant and anesthetic to the urinary tract); and Mel-Maroba (an alternative, reconstructive tonic), and they are elegant. Formula on each package. Send for literature and samples; free. They will also send "A Brief Summary of Hypodermic Medication," and samples of their hypodermic tablets free to physicians on request, naming the Red Back.

---

Neuralgia.—The most frequent causes of neuralgia are a rheumatic or gouty diathesis, blood disorders, malarial conditions, colds and exposure.

In all forms of neuralgia, whether simple or complicated by the anything, the efficacy of Tongaline in relieving the pain of
